# Modified Negative Pressure Wound Therapy After Open-Window Thoracostomy for Empyema

**DOI:** 10.1016/j.atssr.2026.02.027

**Published:** 2026-03-11

**Authors:** Toshio Shiotani, Noriaki Shiraha, Mototsugu Watanabe

**Affiliations:** 1 Department of Thoracic Surgery, NHO Iwakuni Clinical Center, Yamaguchi, Japan

## Abstract

Open-window thoracostomy is an effective surgical procedure for empyema but is often associated with prolonged hospitalization and residual dead space in the thoracic cavity. Although negative pressure wound therapy after open-window thoracostomy is beneficial, its application is challenging in the presence of major air leakage. We present a modified negative pressure wound therapy technique using a suction tube and low-pressure continuous suction that enables treatment regardless of air leakage. This method provides effective drainage, promotes lung reexpansion, and facilitates infection control with reduced medical material costs.

In the management of empyema with fistulas, such as bronchopleural fistula, open-window thoracostomy (OWT) is a highly effective surgical procedure.^1^ Although OWT is effective for infection control, it often results in prolonged hospitalization and residual dead space in the thoracic cavity. Using negative pressure wound therapy (NPWT) in addition to OWT can reduce these complications.^1^^,^^2^ However, in cases with major air leakage, it can be difficult to perform conventional NPWT and control infection. Herein, we report a new NPWT method using suction tubes that enables NPWT regardless of air leakage.

## Technique

**Figure 1:**
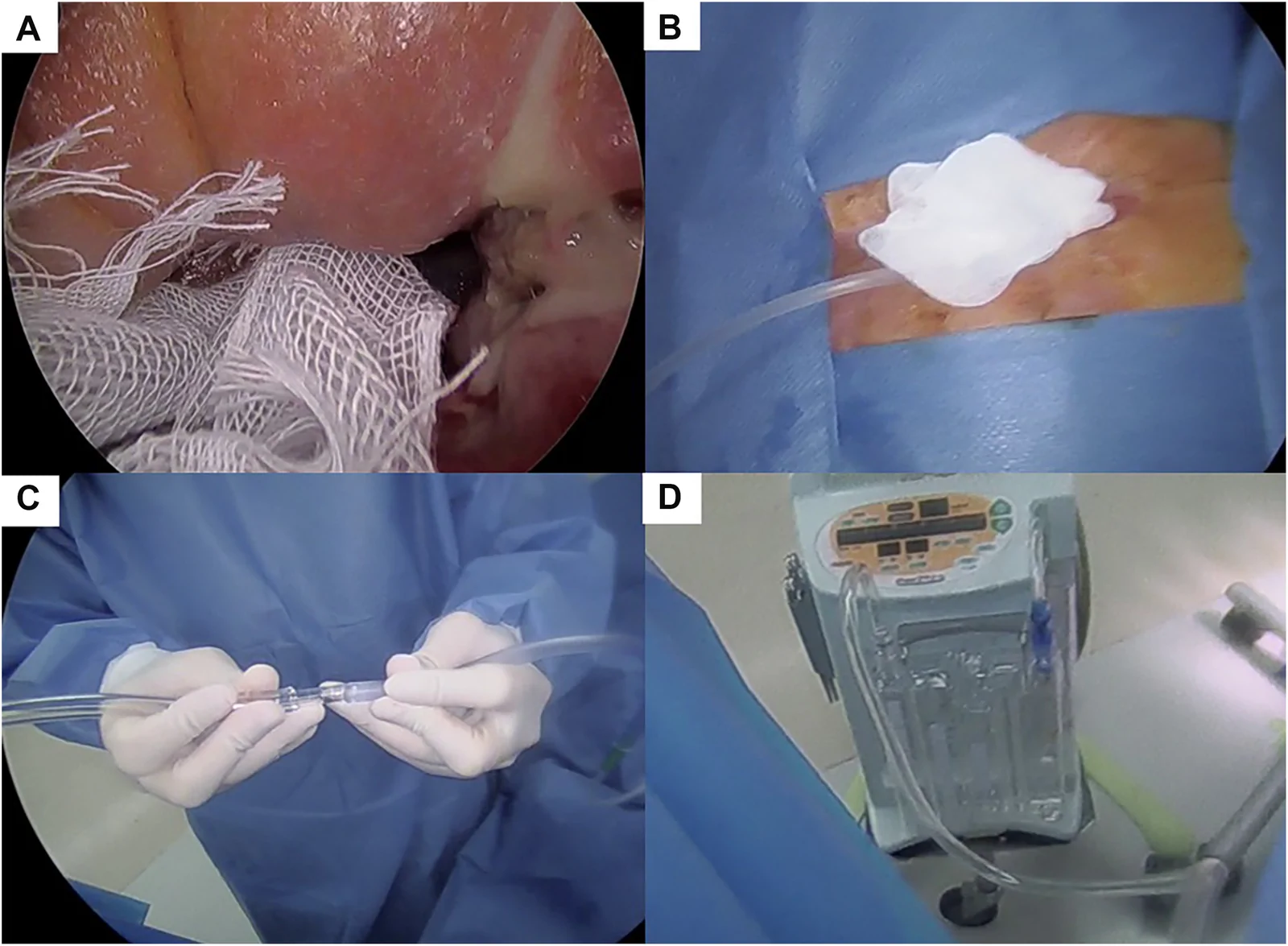
The procedure of the negative-pressure wound therapy after open-window thoracostomy (OWT) for empyema with fistula. (A) Gauze is stuffed into the thoracic cavity after OWT. (B) Insert the suction tube into the cavity with the end positioned between the gauze. The wound is sealed with waterproof, nonbreathable medical dressings. (C, D) The suction tube is connected to the low-pressure continuous suction device and negative pressure is applied.

**Figure 2:**
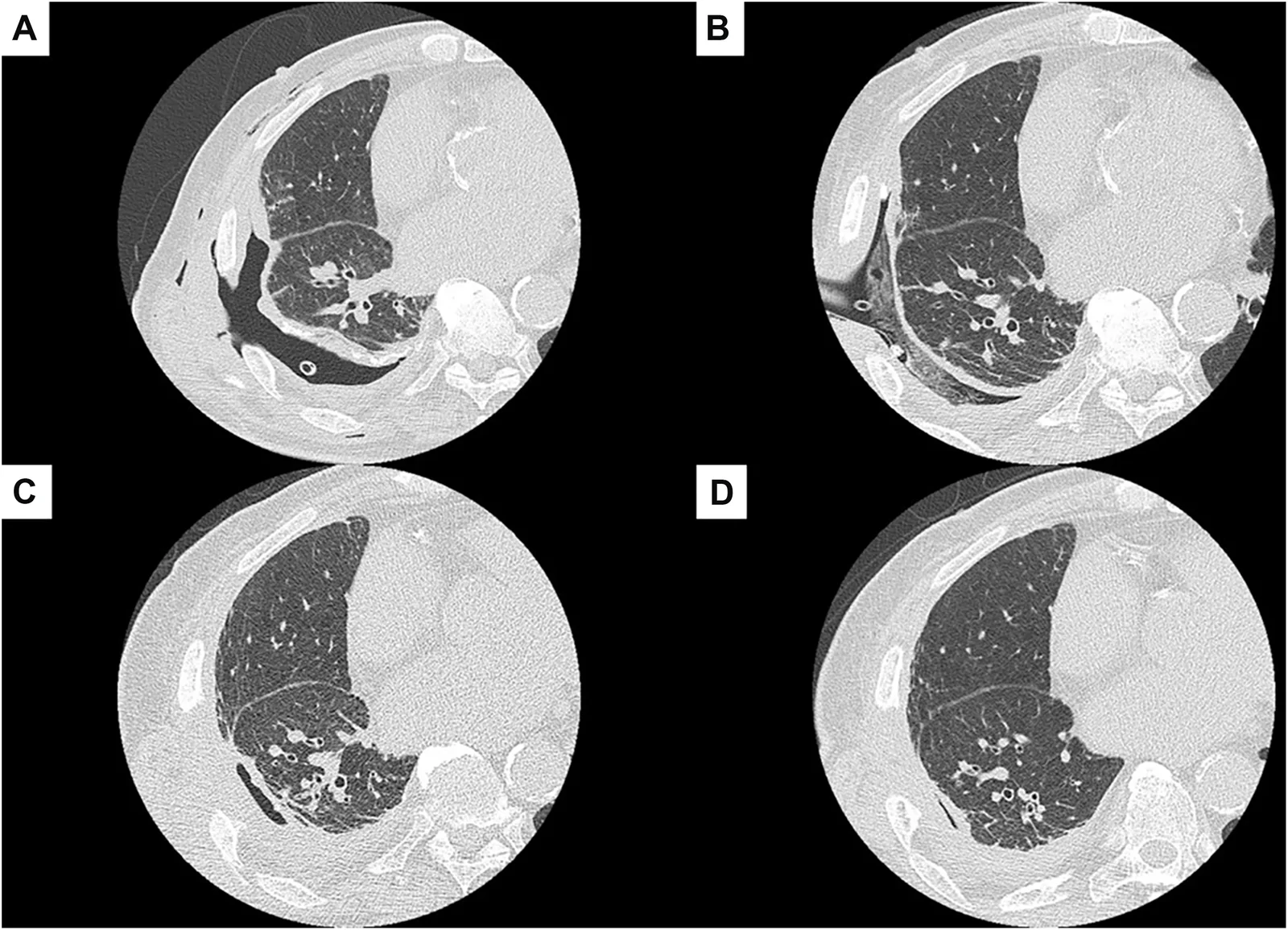
Computed tomography imaging shows the course of a new negative pressure wound therapy (NPWT) using suction tubes after open-window thoracostomy. (A) Compared with before introducing the new NPWT method, (B) marked improvement in lung expansion and thickening of the parietal pleura was observed 22 days after introducing the new NPWT method. (C) A reduction in dead space was observed on day 98. (D) By day 166, the dead space had nearly disappeared, achieving closure of the empyema cavity.

The new NPWT is performed with the patient in the right or left lateral decubitus position. During the change of the gauze in the area where the OWT was performed, a 12F or 14F suction tube is inserted into the gauze. The suction tube is placed within the gauze, ensuring that the tip does not directly contact intrathoracic organs. Subsequently, the wound is sealed with waterproof, nonbreathable medical dressings. The suction tube is connected to a low-pressure continuous suction device, and negative pressure (of approximately 50 hPa) is applied (Figure 1). The fundamental concern during this procedure is to prevent the tip of the suction tube from direct contact with the organs in the thoracic cavity. For several days after starting this treatment, the gauze should be changed daily due to severe contamination and a large amount of pleural fluid in the thoracic cavity. When the infection is controlled and the volume of pleural fluid has decreased, the frequency of gauze changes can be reduced to 2 or 3 times per week. As with conventional NPWT, the dead space in the thoracic cavity gradually decreases over time (Figure 2). The accompanying Video demonstrates our technique for performing the new NPWT after OWT.

## Comment

A useful NPWT management method with a suction tube and a low-pressure continuous suction device was presented. Using this method, no patients died from infection, including pneumonia or empyema, after OWT, except for 1 patient who developed septic shock prior to OWT. Furthermore, this new NPWT can be transitioned to conventional NPWT systems during the treatment course when air leakage is reduced.

This method is particularly useful following OWT when persistent air leaks are present. Although a negative pressure of only 50 hPa is applied, it proves to be sufficient to achieve the reexpansion of the lung.^3^ Indeed, the efficacy of the procedure was demonstrated in a case of bronchopleural fistula with lung collapse treated at our institution. While the possibility of prolonged air leakage due to relatively high negative pressure is a concern, cessation of air leakage has been achieved within 3 months after OWT. Further evaluation with a larger number of cases is required to clarify the optimal negative pressure.

This method also has the potential to serve as a costeffective strategy for infection management and reduction of medical material expenditures. The most common reason for performing OWT treatment is infection control. It is imperative to establish effective drainage mechanisms to ensure the purification of the pleural cavity and to avert the development of aspiration pneumonia caused by purulent pleural effusion. Therefore, strict management, including frequent gauze changes, is necessary during the early phase after OWT. This method applies negative pressure from the early stage, which is considered effective in preventing aspiration pneumonia caused by purulent pleural fluid. The negative pressure allows drainage of pleural fluid that the gauze could not absorb. Furthermore, NPWT can be continued even in cases requiring frequent wound care due to severe contamination, without significant additional costs for medical materials.

There are several limitations of this method. First, this method is performed using gauze rather than a specialized form. Therefore, coverage of the bronchial stump or pulmonary artery stump with nonadhesive gauze may be recommended. Second, achieving a seal may be more difficult compared with conventional NPWT. In cases where negative pressure cannot be maintained, reapplication of the dressing is required. Despite these limitations, this method can represent a valuable and adaptable option for NPWT after OWT, particularly in patients with persistent air leakage.

In conclusion, we present a novel NPWT method using a low-pressure continuous suction device after OWT. This method can be applied regardless of air leakage, reduces medical material costs, and may serve as a useful option after OWT.
